# Subacute Progression of Gait Disturbance and Consciousness Impairment Due to Communicating Hydrocephalus Associated With Vestibular Schwannoma

**DOI:** 10.7759/cureus.89880

**Published:** 2025-08-12

**Authors:** Satoka Yano, Akatsuki Kubota, Mizuho Kawai, Daiki Yashita, Hiroyuki Ishiura, Wataru Satake, Kaoru Yamada, Yuki Shinya, Satoru Miyawaki, Takeshi Iwatsubo, Tatsushi Toda

**Affiliations:** 1 Department of Neurology, The University of Tokyo Graduate School of Medicine and Faculty of Medicine, Tokyo, JPN; 2 Department of Neurology, Okayama University Graduate School of Medicine, Dentistry, and Pharmaceutical Sciences, Okayama, JPN; 3 Department of Neuropathology, The University of Tokyo Graduate School of Medicine and Faculty of Medicine, Tokyo, JPN; 4 Department of Dementia Inclusion and Therapeutics, The University of Tokyo Graduate School of Medicine and Faculty of Medicine, Tokyo, JPN; 5 Department of Neurosurgery, The University of Tokyo Graduate School of Medicine and Faculty of Medicine, Tokyo, JPN; 6 National Institute of Neuroscience, National Center of Neurology and Psychiatry, Tokyo, JPN; 7 National Center Hospital, National Center of Neurology and Psychiatry, Tokyo, JPN

**Keywords:** communicating hydrocephalus, csf dynamics, disorder of consciousness, ventriculoperitoneal shunting, vestibular schwannoma

## Abstract

Patients with vestibular schwannomas (VSs) present with vestibulocochlear nerve dysfunction such as vertigo and tinnitus. VSs occasionally develop communicating hydrocephalus as a complication, which is typically characterized by an insidious progression of symptoms. We report a case of an 84-year-old female patient with a VS who developed gait disturbance and consciousness impairment over a three-week period, ultimately resulting in an inability to walk and communicate. A thorough evaluation ruled out encephalitis and other differential diagnoses. Imaging studies demonstrated findings consistent with communicating hydrocephalus, and a tap test temporarily improved her consciousness disturbances. The patient underwent ventriculoperitoneal shunting and stereotactic radiosurgery (SRS), after which both consciousness and gait disturbances dramatically improved 10 days postoperatively. The subacute development of symptoms due to normal pressure hydrocephalus associated with VSs is rare. Furthermore, to the best of our knowledge, this is the first reported case of severe gait impairment and disturbance of consciousness progressing within a short period. This case highlights the importance of considering communicating hydrocephalus associated with VSs as a differential diagnosis, even in cases of subacute consciousness disturbance. We also discuss the pathophysiology of hydrocephalus in relation to cerebrospinal fluid (CSF) clearance into the extracranial space.

## Introduction

Vestibular schwannomas (VSs) are benign brain tumors originating from Schwann cells in the vestibulocochlear nerve. They most commonly present with symptoms associated with vestibulocochlear nerve dysfunction, such as sensorineural hearing loss and tinnitus. Notably, it has been reported to cause communicating hydrocephalus even when the tumor size is insufficient to induce obstructive hydrocephalus [1]. Hydrocephalus on imaging is present in 15% of VS patients, and of those with hydrocephalus, 23% (representing 3.5% of the entire cohort) exhibit communicating hydrocephalus [2]. The exact mechanism underlying the development of communicating hydrocephalus in patients with VSs remains unclear.

Here, we present a case of an 84-year-old woman with a VS who rapidly developed gait disturbance and consciousness impairment over a three-week period due to communicating hydrocephalus associated with the tumor. The patient showed significant improvement following ventriculoperitoneal shunting and stereotactic radiosurgery (SRS). To the best of our knowledge, this is the first case demonstrating severe and reversible consciousness impairment in a subacute course due to VS. This case report aims to describe a rare subacute presentation of communicating hydrocephalus with VS, highlighting the diagnostic challenge and the importance of early intervention. Furthermore, this case offers a valuable opportunity to explore the underlying pathophysiological mechanisms, including molecular pathways involved in the clearance of substances from the cerebrospinal fluid (CSF) to the extracranial space.

## Case presentation

An 84-year-old female patient presented with a three-year history of progressive hearing loss in her right ear, while remaining independent in daily activities, with no history of alcohol consumption and no significant medical or family history of note. Three weeks prior to admission, she began experiencing unsteadiness while walking, frequently falling, and became unable to manage her medications independently. Nine days before admission, she developed urinary incontinence and exhibited abnormal behaviors, such as drinking water after gargling. Five days before admission, she became increasingly drowsy, with speech limited to single words and difficulty with oral intake. She was subsequently admitted to our hospital for further evaluation.

**Figure 1:**
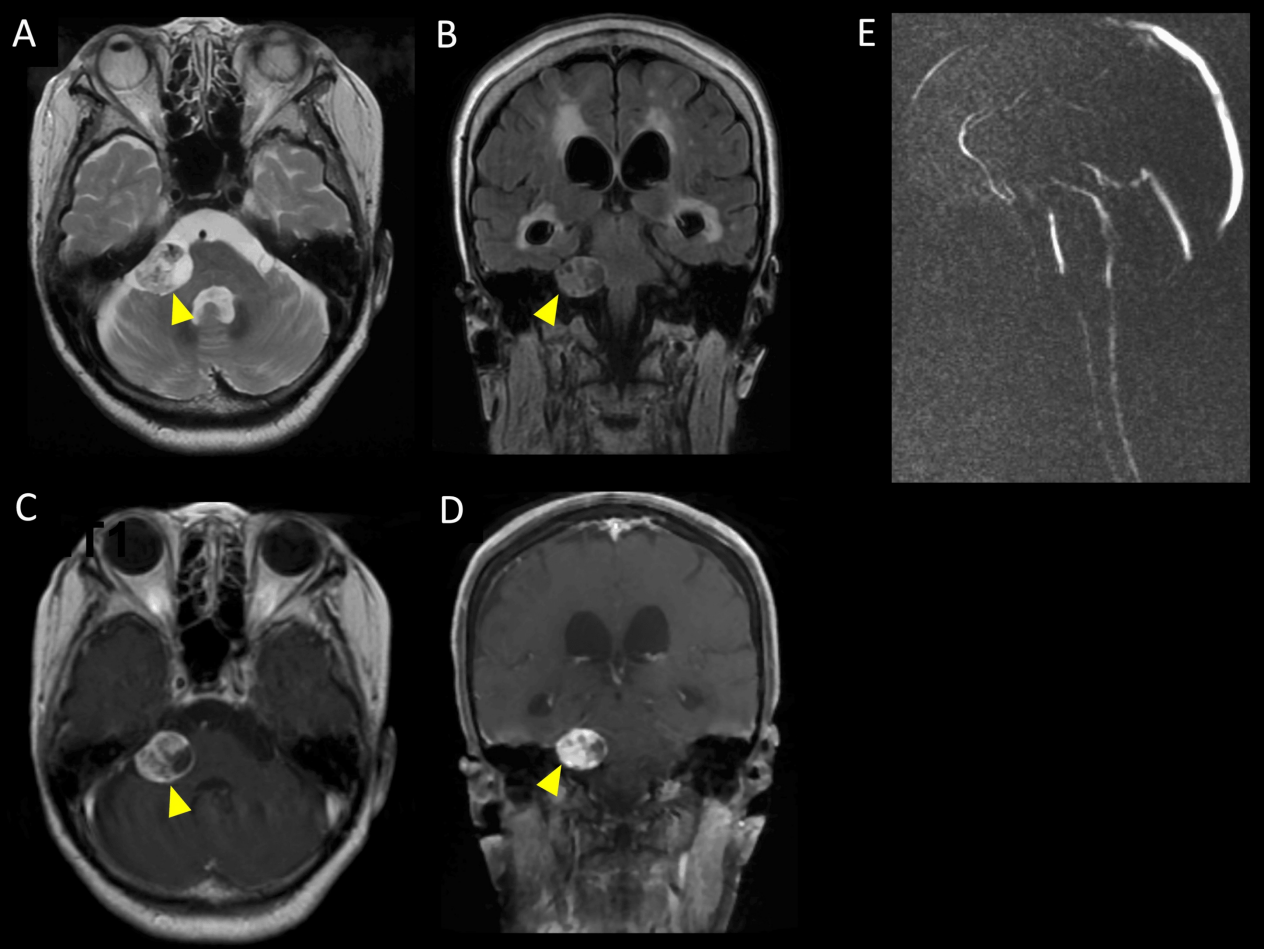
Brain MRI findings on admission VS: vestibular schwannoma; CSF: cerebrospinal fluid; MRI: magnetic resonance imaging; T2WI: T2-weighted imaging; FLAIR: fluid-attenuated inversion recovery (A) Brain MRI reveals a 22 mm VS with a mixture of moderately high- and low-intensity signals in the right cerebellopontine angle on axial T2WI (a yellow arrowhead). (B) The coronal FLAIR image shows that the tumor is compressing the brainstem to the left. (C, D) Gadolinium-enhanced T1-weighted imaging shows enhancement of the tumor and ventricular enlargement (a yellow arrowhead). (E) Cine MRI shows CSF flow from the fourth ventricle through the cerebral aqueduct into the third ventricle

**Figure 2:**
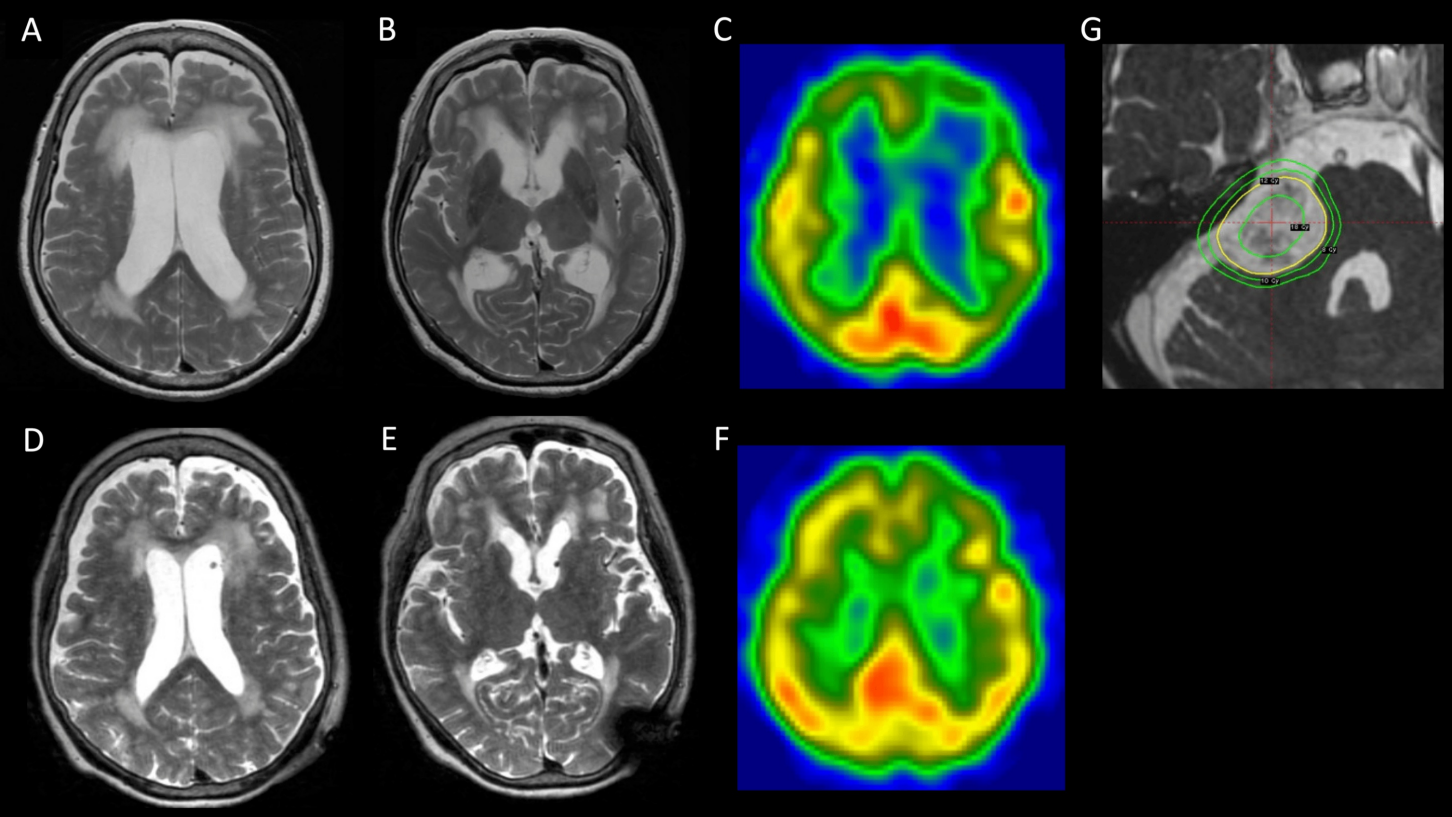
Brain MRI and 123I-IMP SPECT findings before and after ventriculoperitoneal shunting MRI: magnetic resonance imaging; ^123^I-IMP SPECT: N-isopropyl-p-^123^I-iodoamphetamine single-photon emission computed tomography; T2WI: T2-weighted imaging; SRS: stereotactic radiosurgery (A, B) Brain MRI on axial T2WI at the time of admission (Evans index, 0.34). (C) ^123^I-IMP SPECT at the time of admission shows hypoperfusion in the bilateral frontal lobes. (D, E) Brain MRI on axial T2WI seven days after ventriculoperitoneal shunting shows improved ventricular enlargement (Evans index, 0.28). (F) ^123^I-IMP SPECT 25 days after ventriculoperitoneal shunting shows improvement in bilateral frontal hypoperfusion. (G) SRS protocol using the Leksell Gamma Knife shows a marginal dose of 12 Gy and a central dose of 24 Gy

On neurological examination at the time of admission, the patient showed signs of consciousness impairment (Glasgow Coma Scale (GCS) score: E3V3M5), frontal lobe signs such as palmar grasp reflex and snout reflex, and a positive left Babinski sign. Her score in the revised version of Hasegawa’s Dementia Scale (HDS-R) was 1/30 (immediate recall only), and neither the Frontal Assessment Battery (FAB) nor the Trail Making Test (TMT) could be performed [3]. The patient was unable to stand. Laboratory blood test results revealed no evidence of viral, fungal, or mycobacterial infections. The levels of vitamins B1 and B12 were within normal limits, and there were no elevations in the levels of ACE, tumor markers, or the soluble IL-2 receptor. Tests for autoimmune diseases and encephalitis, including anti-thyroid peroxidase (TPO) antibody, anti-N-methyl-D-aspartate receptor (NMDAR) antibody, paraneoplastic antibodies, and anti-glutamic acid decarboxylase (GAD) antibody, all showed negative results. CSF analysis revealed a significant increase in protein levels (209 mg/dL), but otherwise, the results were normal (initial pressure, 11.5 cmH2O; cell count, 0/µL; Glu, 116 mg/dL; IgG index, 0.59; CSF cytology, Class I), and phosphorylated tau levels in the CSF were below 25 pg/mL. Brain magnetic resonance imaging (MRI) revealed a 22 mm VS with partial intratumoral hemorrhage in the right cerebellopontine angle and ventricular enlargement (Evans index 0.34) (Figures 1A-1D, 2A-2B). Cine MRI confirmed CSF flow from the fourth ventricle through the cerebral aqueduct into the third ventricle (Figure 1E). N-isopropyl-p-^123^I-iodoamphetamine single-photon emission computed tomography (^123^I-IMP SPECT) demonstrated hypoperfusion in the bilateral frontal lobes (Figure 2C). The patient’s consciousness deteriorated further, with her GCS score dropping to E2V3M5 on day 3 after admission. 

Given these findings, communicating hydrocephalus associated with the VS was suspected. Since CSF communication was preserved and there were no findings suggestive of increased intracranial pressure, a lumbar tap test was deemed feasible and was performed on hospital day 4, removing 30 mL of CSF, resulting in a temporary improvement in consciousness (GCS score: E3V4M6). However, within 24 hours, her condition deteriorated again to a GCS score of E2V3M5. A ventriculoperitoneal shunt was placed 10 days after admission. A programmable valve was selected for its ability to allow postoperative pressure adjustments based on clinical response and was initially set at 11.0 cmH2O. No postoperative complications occurred following ventriculoperitoneal shunting placement, including common shunt-related complications such as infection, malfunction, or overdrainage. Postoperatively, her consciousness level progressively improved from the day after surgery, and after three days, her spontaneous speech improved, and her speech became fluent. On postoperative day 11, her sense of orientation improved, her level of consciousness improved to a GCS score of E4V5M6, and her HDS-R score increased to 27/30. Her gait disturbance also improved; she was able to stand on postoperative day 4 and walk independently by postoperative day 10. The left Babinski sign and frontal lobe signs resolved. Follow-up brain MRI demonstrated improvement in hydrocephalus (Evans index 0.28) (Figures 2D-2E), and ^123^I-IMP SPECT showed improvement in bilateral frontal hypoperfusion (Figure 2F). Given the patient's advanced age, SRS using the Leksell Gamma Knife (Elekta Instruments, Stockholm, Sweden) was selected for the treatment of her VS, with a marginal dose of 12 Gy and a central dose of 24 Gy, as an alternative to surgical resection (Figure 2G). After the procedure, she was transferred to a rehabilitation hospital for further care. In accordance with our standard practice in the SRS clinic, follow-up has been conducted, including routine brain MRI every six months following treatment. During the four-year follow-up period to date, no remarkable findings have been observed on neurological examination, and there has been no evidence of hydrocephalus exacerbation on imaging studies.

## Discussion

Here, we present a case of subacute progression of consciousness impairment due to communicating hydrocephalus associated with a VS. Results of laboratory blood tests and CSF analysis revealed no evidence of infections, autoimmune, inflammatory, or neoplastic diseases. Imaging studies confirmed the presence of a VS without obstruction of CSF circulation, and the positive response to a lumbar tap further supported this diagnosis. In contrast, the subacute progression of symptoms in this condition is rare and atypical. Whereas in some reports, the shortest disease duration is six weeks to two months [1,4], cases with severe symptoms such as impaired consciousness and gait disturbance, as observed in this case, are not described. There has been a reported case of a VS patient presenting with an acute onset and severe consciousness disturbance; however, it involved obstructive hydrocephalus, not communicating hydrocephalus [5]. To the best of our knowledge, there have been no reported cases of communicating hydrocephalus associated with VSs in which symptoms, including gait disturbance and consciousness impairment, progressed to such a severe degree within a short period after onset. This rarity posed significant challenges in establishing a diagnosis.

In cases of communicating hydrocephalus associated with VSs, ventriculoperitoneal shunting is expected to improve symptoms regardless of the severity before treatment [1]. There are reports of combined tumor resection with ventriculoperitoneal shunting; however, SRS is a primary therapeutic modality for small- to medium-sized sporadic VSs, offering significant advantages, including excellent tumor control, low facial nerve toxicity, and minimal invasiveness [6,7]. In this particular case, given the patient’s advanced age, SRS was applied. Note that the phenomenon of hydrocephalus secondary to tumor protein leakage post-SRS has been reported in the literature [7]. Therefore, diligent follow-up for the potential development of hydrocephalus following SRS is warranted for several years [8]. In accordance with our standard practice in the SRS clinic, brain MRI has been routinely performed every six months following treatment, with no evidence of hydrocephalus exacerbation observed during the follow-up period. This case highlights the potential efficacy of combining ventriculoperitoneal shunting with single-session SRS as a therapeutic approach to managing communicating hydrocephalus associated with VSs, particularly in elderly patients in whom invasive surgery should be avoided. Given the potential for significant symptom improvement following treatment, as experienced in this case, it is crucial to consider communicating hydrocephalus associated with VSs as a possible cause of subacute progression of consciousness impairment to ensure timely intervention.

VSs can lead to communicating hydrocephalus [1]. Although the underlying mechanism remains unclear, it has been proposed that intratumoral hemorrhage or an increase in protein levels in the CSF may lead to impaired CSF absorption [9]. The clearance of waste products and substances from CSF remains largely unexplored. Recent studies have proposed the glymphatic system as an efficient mechanism for the clearance of metabolic waste from the brain parenchyma [10]. The glymphatic system is involved in the flow of CSF through the perivascular space, which surrounds blood vessels in the brain. This flow occurs along the arterial perivascular spaces, where CSF enters and is driven by arterial pulsation, before mixing with interstitial fluid in the brain tissue. The mixed fluid is then cleared through the venous perivascular spaces, effectively transporting metabolic waste and soluble proteins out of the brain. Furthermore, functional lymphatic vessels have been identified in the dura mater as an exit route for CSF drainage, with CSF being absorbed through these vessels and eventually reaching deep cervical lymph nodes [11,12].

In the current case, marked protein level elevation was observed in the CSF, but it is known that elevated protein levels in the CSF do not always correlate with the development of normal pressure hydrocephalus [13]. It is conceivable that changes in the type or composition of leaked proteins, rather than their total amount, could affect CSF absorption efficiency. The glymphatic system is affected by various factors, including vascular pulsations [14], the perivascular localization of aquaporin-4 [15], and perivascular macrophages [16]. Although much remains unknown about the regulatory factors for the lymphatic system, which serves as a CSF outflow pathway, it is plausible that the types of proteins whose levels are elevated in the CSF may potentially affect CSF clearance mechanisms. From this perspective, the present case provides valuable insights into the mechanisms of CSF clearance and its regulatory processes, contributing to a deeper understanding of this complex system.

## Conclusions

VSs can cause communicating hydrocephalus, leading to the subacute progression of consciousness impairment, as demonstrated in this case. In cases of communicating hydrocephalus associated with VSs, the combination of ventriculoperitoneal shunting and SRS offers a significant potential for symptom improvement while achieving excellent tumor control. Our experience underscores the need for a high index of suspicion for communicating hydrocephalus in patients with VS presenting with subacute progression of consciousness impairment, ensuring that timely intervention is not delayed.
